# An Updated View of the Effect of Probiotic Supplement on Sports Performance: A Detailed Review

**DOI:** 10.1007/s13668-024-00527-x

**Published:** 2024-03-12

**Authors:** Miray Nur Aykut, Esma Nur Erdoğan, Menşure Nur Çelik, Murat Gürbüz

**Affiliations:** 1 Department of Nutrition and Dietetics, Trakya University, Edirne, Turkey; 2 Department of Nutrition and Dietetics, Ondokuz Mayıs University, Samsun, Turkey

**Keywords:** Probiotic, Sports performance, Intestinal barrier function, Aerobic capcacity

## Abstract

**Purpose of Review:**

Modulation of the host microbiota through probiotics has been shown to have beneficial effects on health in the growing body of research. Exercise increases the amount and diversity of beneficial microorganisms in the host microbiome. Although low- and moderate-intensity exercise has been shown to reduce physiological stress and improve immune function, high-intensity prolonged exercise can suppress immune function and reduce microbial diversity due to intestinal hypoperfusion. The effect of probiotic supplementation on sports performance is still being studied; however, questions remain regarding the mechanisms of action, strain used, and dose. In this review, the aim was to investigate the effects of probiotic supplements on exercise performance through modulation of gut microbiota and alleviation of GI symptoms, promotion of the immune system, bioavailability of nutrients, and aerobic metabolism.

**Recent Findings:**

Probiotic supplementation may improve sports performance by reducing the adverse effects of prolonged high-intensity exercise.

**Summary:**

Although probiotics have been reported to have positive effects on sports performance, information about the microbiome and nutrition of athletes has not been considered in most current studies. This may have limited the evaluation of the effects of probiotic supplementation on sports performance.

## Introduction

Probiotic is derived from the Greek word “pro bios” meaning “vitality,” as opposed to “antibiotic” meaning “anti-vitality.” Although the presence of acid-producing bacteria in fermented dairy products was mentioned by Metchnikoff in 1907, the concept of “probiotic” was first used by Lilly and Stillwell in 1965. Later, this concept was defined by Fuller in 1989 as “microbial reinforcement that increases the stability of the host’s gut” [1]. Because of the rapid increase in research on this subject, the need for a common “probiotic” definition has emerged. The World Health Organization (WHO) and United Nations Food and Agriculture Organization (FAO) have defined probiotics as “live microorganisms that provide health benefits to the host when administered in adequate amounts” [2]. A sufficient amount to be administered should be more than 10^6^ colony-forming units (CFU)/mL of live microorganism population for the aforementioned health benefits [3]. In addition, live microorganism species must be safe, have a high tolerance to low pH conditions and bile acids, promote intestinal colonization, and not be vectors of antibiotic resistance genes [4, 5].

The potential health benefits of probiotics may differ according to the microorganism strains [3]. Probiotics may generally produce health effects through the synthesis of beneficial molecules such as short-chain fatty acids (SCFAs), bacteriocins, vitamin K, and vitamin B complex; secretion of protein/peptide structures such as antimicrobial peptides and secretory IgA from the intestines; reduction of pathogenic toxins; protection of epithelial barrier integrity; and immune system regulation [6, 7]. Research on the health effects of probiotics is constantly increasing, and probiotics are thought to exert health effects primarily by modulating host microbiota [8].

In athletes, the number of beneficial microorganisms and microbial diversity has been shown to be greater than in their sedentary counterparts. However, prolonged high-intensity exercise is associated with reduced microbial diversity due to intestinal hypoperfusion [10]. Probiotic supplementation may be an effective strategy to offset the negative effects of prolonged high-intensity exercise [11, 12]. Specifically, probiotics have been associated with reduced gastrointestinal symptoms and infection susceptibility, and may improve performance by improving muscle energy production capabilities, muscle mass and strength, and aerobic capabilities [11, 13, 14]. However, the omission of information regarding the microbiomes of athletes and their diets at the beginning of existing studies hinders the interpretation of the outcomes obtained. Furthermore, probiotic supplementation has also been reported to have no effect on sports performance [15, 16]. Exercise intensity, type of probiotic, dose used, and duration of treatment may have contributed to the heterogeneous results. The aim of our study was to examine the direct and indirect effects of various probiotic interventions on sports performance in line with current literature.

## Definitions and Types of the Probiotics

The use of probiotics in human history dates back to 7000 BC to ferment milk and fruits for long-term preservation. In ancient Greece, fermented foods were used in medical treatment for antiseptic, diuretic, and sedative purposes. Turks tried to prevent and treat gastrointestinal (GI) symptoms by consuming yogurt during the Karakhanid period [17]. *Lactobacillus* spp. were first discovered by Pasteur in 1856, after the early modern period. Although beneficial bacterial species were defined by Metchnikoff, the concept of probiotics was first defined by Lilley and Stillwell in 1965 as “microbes that stimulate the growth of other microorganisms.” In 1989, Fuller defined probiotics as “microbial supplements that increase the stability of the host’s gut.” In 2001, WHO and FAO updated the definition of probiotics based on the latest evidence as “live microorganisms that provide health benefits to the host when applied in adequate amounts” [18].

**Fig. 1:**
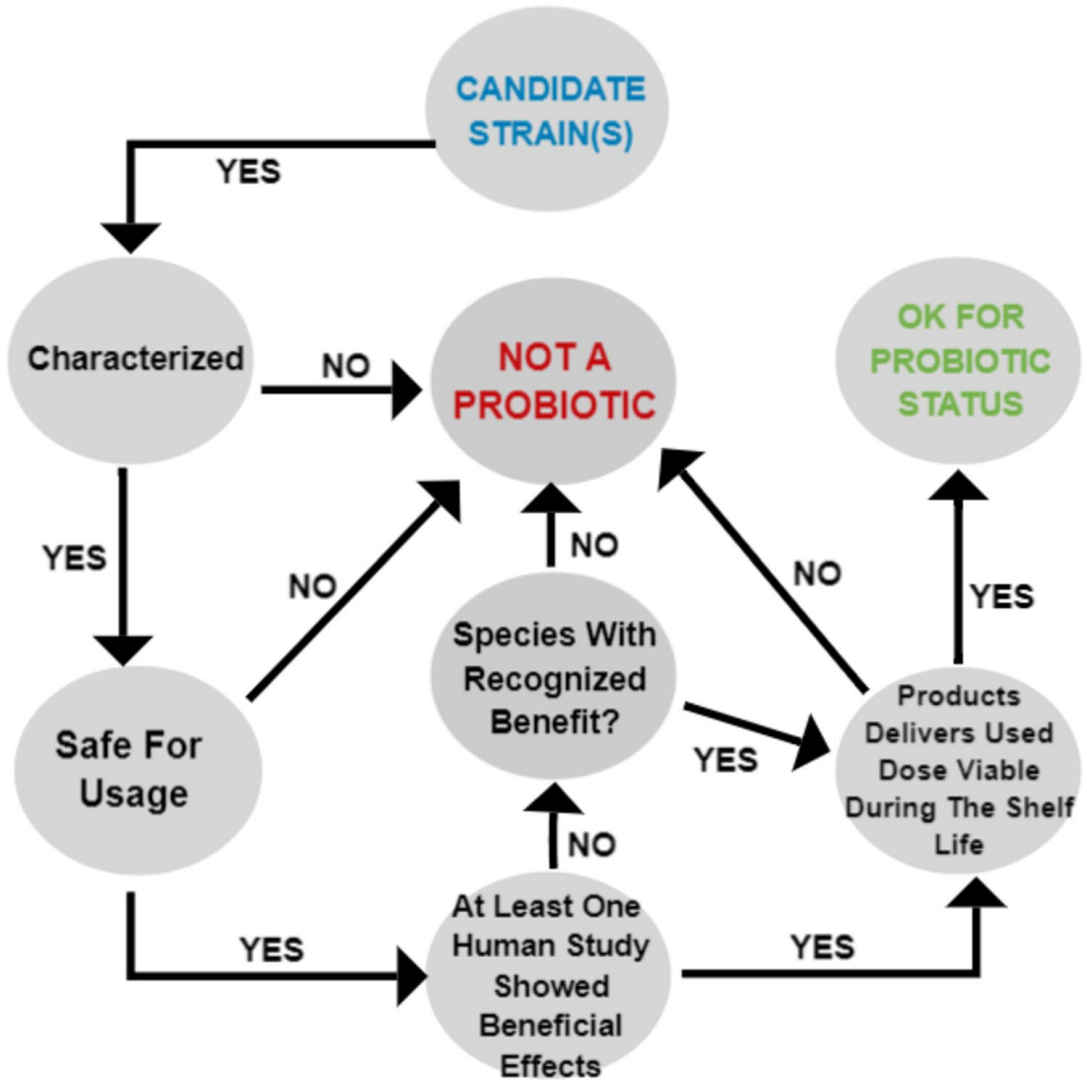
Decision tree used to determine a candidate probiotic

Many microorganism species have been defined to date, and the range of microorganisms claimed to have probiotic properties is gradually expanding owing to improved culture methods and sequencing techniques. However, according to the definition of probiotics by the International Scientific Association for Probiotics and Prebiotics (ISAPP), when taken by the host, probiotics must be alive, create health benefits, and be administered in effective doses [19]. In a significant number of studies, the microorganism survival and colonization in the host GI tract have been considered to determine probiotic efficacy [3, 5]. However, ISAPP states that probiotics do not require conditions such as survival, colonization, anti-pathogenic properties, or balancing of the host microbiota [19]. The decision tree shown in Fig. 1 is used to name the newly discovered microorganism strain as a probiotic. According to this tree, (i) probiotic strains should be named in accordance with the International Nomenclature Code, (ii) the health effects of probiotics should be supported by at least one clinical study, and (iii) microorganisms should remain alive at the effective dose until the expiration date of the probiotic product and be safe [20]. Many strains of *Lactobacillus* spp., *Bifidobacterium* spp., and some yeasts have a history of safe use, which does not pose a significant safety concern in the diet. However, the European Food Safety Authority (EFSA) has recommended the evaluation of the Qualified Presumption of Safety (QPS) list published for healthy populations and safety reports of in vivo studies on the consumption of food and dietary supplements containing probiotics [20, 21].

Probiotics can be consumed as food or as dietary supplements. However, the terms probiotic food and fermented food are often confused by consumers. The health benefits of fermented foods such as pickles, yogurt, cheese, and vinegar are supported in some epidemiological studies [22]. For example, EFSA has validated the health claim that yogurt can improve lactose tolerance in lactose intolerant individuals (EFSA, 2010). In a cohort study, consumption of fermented soy products was linked to a reduction in cardiovascular disease risk [23]. Despite their health benefits, not all fermented foods meet probiotic definition criteria. While the types of microorganisms in fermented foods may differ according to the producer or production region, the type of microorganisms in probiotic foods should be defined. Some probiotic foods can be produced by adding probiotics to the production process of fermented food [19]. Although *Lactobacillus*, *Bifidobacterium*, and *Saccharomyces* cultures are frequently used in this process, research on the determination of candidate probiotic species, called new-generation probiotics, has been increasing in recent years. New-generation probiotics may be important for the development of live biotherapeutic products for prophylactic and therapeutic purposes.

## In Vitro and Animal Studies on Benefaction of Probiotics for Sports Performance

Probiotics can exert bioactivity by inhibiting pathogens by secreting antimicrobial substances such as bacteriocins and increasing intestinal acidity through SCFA synthesis [24, 25]. Probiotics can also contribute to the development of the epithelial barrier by increasing the expression of tight junctions such as claudin-1 and occludin, increasing the secretion of antimicrobial compounds such as defensin, and stimulating the activation of lymphocytes in gut-associated lymphoid tissue [26, 27]. Thus, researchers have aimed to reshape the gut microbiome with probiotic supplementation. However, clinical evidence regarding the effects of probiotics on the intestinal microbial balance is limited. Claims regarding the sports performance of probiotics have mostly been derived from in vitro and animal studies.

Probiotics may affect sports performance through several local or systemic effects, such as regulating the immune response, increasing resistance to infections, reducing depressive symptoms, and maintaining skeletal muscle health [28–30]. According to Vargoorani et al. *Lactobacillus casei* extracellular vesicle can reduce inflammation in Caco-2 cells by decreasing Toll-like receptor (TLR)-9 expression and Interferon (IFN)-γ levels, and increasing Interleukin (IL)-4 and IL10 levels [31]. Similarly, *Weissella cibaria* JW15 strain inhibited inflammation by reducing IL1ß, IL6, and Tumor necrosis factor (TNF)-α levels in Lipopolysaccharide (LPS)-induced RAW 264.7 cells [32]. Evidence regarding the ability of probiotics to inhibit the inflammatory response has been reported by several meta-analyses [33, 34•]. Additionally, probiotics may reduce susceptibility to certain infections, such as upper respiratory tract infections (URTIs). For example, the *Lactobacillus plantarum* strain inhibited *Streptococcus pyogenes*, which frequently infects the respiratory tract, in human lung alveolar epithelial cell culture [35]. Furthermore, *L. casei* 431 and *L. fermentum* PCC can beneficially regulate penicillin-induced imbalance in the URT microbial compositional structure of experimental mice and thus modulate the immune response [36].

The influence of gut microbiota via the gut-brain axis on the psychological state and brain function of the host has become a popular topic in recent years. In animal models, intestinal dysbiosis has been associated with an abnormal stress response and neuroinflammation in the host [37, 38]. In particular, *Lactobacillus* spp. have been shown to prevent dysbiosis by regulating intestinal serotonin metabolism, which leads to a reduction of abnormal behavior in experimental animals [39, 40]. More recently, the health implications of the gut microbiome have been extended beyond the gut-brain axis. Gut health has been linked to muscle health, which is termed the gut-muscle axis. In patients with inflammatory bowel disease, decreased levels of *Firmicutes* and *Bacteroides*, and increased levels of *Enterobacteriaceae* were observed. This shift in microbial community may be associated with decreased muscle function and cachexia [41]. Probiotic supplementation can limit decreased muscle function and cachexia in rodents [42, 43]. In rodent models, probiotics have been reported to exert their effects on muscle mass and function through protein bioavailability, preservation of muscle strength and endurance, reduction of fatigue markers, and an increase in muscle glycogen stores [44, 45].

## Mechanisms of Action of Probiotics on Sports Performance

Athletes are at risk for various diseases because of training, travel, insufficient rest, and malnutrition [46]. For example, GI symptoms and endotoxemia are frequently reported, especially in long-distance athletes such as marathons and triathlons [47, 48]. Although low- and moderate-intensity exercise reduce physiological stress and improve immune function, high-intensity prolonged exercise suppresses immune function from a few hours to several days [49, 50]. This is called the “open window period,” in which susceptibility to infections, such as URTIs, increases in athletes. Probiotics may be effective in infection control [13, 51]. Probiotics can also increase the amount of glycogen in the liver and skeletal muscles, and increase the absorption of amino acids, which are important for protein synthesis, such as branched-chain amino acids (BCAAs) and glutamine [52–54]. Moreover, the gut microbiota can improve muscle strength and function through the intestinal axis action [55–57]. Therefore, the effectiveness of probiotics in improving physical performance has become a focus of research in recent years.

### Gastrointestinal System and Modulation of Intestinal Microbiota

Modulation of the intestinal microbiota and various metabolites, such as short-chain fatty acids, can reduce intestinal epithelial barrier permeability and production of inflammatory cytokines. Thus, it reduces GI disturbances, delays fatigue symptoms, increases skeletal muscle mass and function, and enhances athletic performance [58–61]. Furthermore, short-chain fatty acids, such as butyrate, increase the proportion of oxidative fibers by stimulating peroxisome proliferator-activated receptor-1/ (PGC-1) [62]. This increase may positively affect skeletal muscle endurance and exercise performance [63].

Probiotics may help restore impaired intestinal microbiota and support microbiota under stress conditions [64, 65]. The frequency and severity of GI symptoms were reduced by probiotic supplementation and microbiota modulation in athletes [66••, 67]. These symptoms are caused, in part, by decreased oxygen and nutrient supply to enterocytes due to intestinal hypoperfusion, decreased mucus layer thickness, increased intestinal permeability, and bacterial translocation into the bloodstream [68]. Increased levels of inflammatory cytokines and bacterial endotoxins were observed as a result of intestinal permeability. Runners with high endotoxin levels after a race are four times more likely to experience GI symptoms than those with low endotoxin levels [69]. GI symptoms can significantly reduce the athletic capacity and performance. Therefore, new strategies that specifically focus on reducing these symptoms should be developed. Dietary strategies have the potential to increase the physical comfort of athletes and reduce the risk of GI disturbances [14]. Probiotic supplementation, an important dietary strategy, can help improve the intestinal barrier, prevent endotoxemia, and alleviate the inflammatory response [70]. Probiotics are effective and safe in preventing and treating GI disturbances caused by intense exercise, thereby improving physical performance [46, 71].

### Immune System Modulation

In addition to modulating the intestinal microbiota, probiotics may regulate the mucosal immune response, enhance macrophage activity, and modulate the expression of genes associated with macrophage activity [72]. Furthermore, probiotics have been shown to reduce the expression of nuclear factor kappa β (NF-κβ) and proinflammatory cytokines by interacting with TLRs [73, 74]. Moreover, anti-inflammatory cytokines, immunoglobulin levels, immune cell proliferation, and production of proinflammatory cytokines by T cells can also be modulated by probiotic supplementation [75, 76]. Probiotics can affect sports performance by modulating the immune system and by improving fatigue indicators and muscle soreness. Moreover, it may indirectly contribute to sports performance by preventing immunosuppressive effects and URTIs caused by intense exercise.

In contrast to recreational and moderate exercise, intense exercise increases the synthesis of proinflammatory cytokines, such as IL-1, IL-6, and TNF-α [77–80]. Changes in salivary IgA levels after exercise may be associated with a higher risk of infection among athletes. However, the literature on salivary IgA is conflicting [81, 82]. Some authors have stated that saliva quality should be evaluated by measuring the total protein concentration as well as the salivary IgA concentration [82]. Moreover, after intense and prolonged exercise, a decrease in the frequency and function of acquired immune cells, such as lymphocytes, has been detected in peripheral blood [83, 84]. This may cause an increase in infection susceptibility and, therefore, a decrease in sports performance. Probiotic supplementation may have an important role in improving factors that may adversely affect sports performance, such as fatigue, pain, mood changes, and concentration disorders after exercise through cytokine modulation. It may also contribute to sports performance indirectly by improving lung performance during and after URTIs or by preventing immunosuppressive effects [78•].

### Bioavailability of Nutrients

Several factors can lead to GI disturbances during endurance exercise, including splanchnic oxidative stress, hypoxia, mechanical stress, exercise-induced hyperthermia, and carbohydrate malabsorption [85, 86]. Reduction in carbohydrate absorption is considered a limiting factor for performance in endurance exercises lasting longer than 60 min. According to some researchers, the ability of probiotics to maintain intestinal integrity may affect sports performance by improving the absorption of carbohydrates and amino acids during prolonged exercise [66••, 87]. It is one of the main claims that probiotic supplementation provides an increase in proteolytic activity by optimizing intestinal microbiota composition [88]. Second, probiotic supplementation increases the absorption of amino acids in vegetable proteins, which are considered low-quality protein sources, and BCAAs, which are important for protein cycling [12, 89]. Thus, the use of probiotics may increase protein bioavailability. However, research on the potential of probiotics in improving nutrient metabolism in relation to exercise remains limited.

Another topic of interest in the relationship between probiotics and nutrient absorption is inorganic iron supplementation. Iron is important for oxygen transport, mitochondrial energy production, and cellular immune response. Physical performance and adaptation to training may be negatively affected by iron deficiency [90]. Increased iron absorption may be a strategy for improving the iron status and avoiding adverse effects from the use of traditional high-dose iron supplements. According to meta-analysis findings, iron absorption was increased in humans supplemented with *L. plantarum* 299v alone [91]. Although the impact of probiotics apart from *L. plantarum* 299v on iron absorption remains unclear, it is still a topic of investigation. Furthermore, the consequences of probiotic supplementation on biochemical markers of iron status, rather than iron absorption, should also be reported in comprehensive studies.

### Aerobic Capacity

Changes in intestinal microbial flora have been suggested to affect hematopoiesis and erythropoiesis by affecting the levels of circular SCFA [93, 94]. Hematopoiesis is suppressed in conditions that cause intestinal microbiome imbalance such as obesity, malnutrition, and antibiotic use [93]. Therefore, the relationship between dysbiosis and hematological problems, such as anemia and neutropenia, has been emphasized in recent studies [95, 96]. Because of the possible improvements in microbial flora and the synthesis of some metabolites in the gut, such as SCFAs, the possibility that probiotic supplementation can increase aerobic performance and endurance by accelerating erythropoiesis has emerged. Aerobic capacity is measured directly using maximal physical workrate tests or indirectly using some equations and is expressed as maximal oxygen uptake (VO2_max_) [97]. Although probiotics have been reported to improve endurance by increasing VO2_max_ in a few studies, some researchers have not confirmed this effect in athletes [98••, 99]. Furthermore, the correlation between VO2_max_ and sports performance is weak in highly trained athletes, owing to compensatory factors [97].

## Clinical Evidence on the Effect of Probiotic Supplementation on Sports Performance

As shown in Table 1, the potential effects of probiotic supplementation on sports performance have been reported in several randomized controlled trials (RCTs). Single- or multi-strain supplements at the level 10^8^–10^11^ CFU/day were used in these studies. In recent studies, sports performance has generally been associated with indicators of fatigue, physical performance, aerobic capacity, carbohydrate and protein bioavailability, inflammatory responses, URTIs, GI symptoms, and psychological status. Researchers have tried to determine the effect of probiotic supplementation on sports performance, mostly directly by measuring physical performance or indirectly by evaluating the inflammatory response and GI symptoms. There is some evidence to suggest that probiotic supplementation modulates the inflammatory responses and reduces the severity of GI symptoms. However, further studies with larger groups are needed to clarify whether probiotic supplementation enhances physical performance.

**Table 1:** Current studies on the effect of probiotic supplementation on sports performance

Strain and daily dose	Participants and method	General effects	Results of the current research	References
2.0 × 10^10^ CFU/day *Lactobacillus paracasei* PS23	20–40 years old, physically active people (*n* = 105) 6 weeks, 2 capsules/day	Fatigue indicators Physical performance Inflammatory response	Probiotic supplementation prevented loss of strength and exercise performance after muscle damage, and improved inflammatory markers	[113]
1.0 × 10^11^ CFU/day multi-strain *L. plantarum*, *L. casei*, *L. rhamnosus*, *Bifidobacterium breve*, *L. acidophilus*, *B. longum*, *B. bifidum*, *B. infantis*, *L. helveticus*, *L. fermentum*, *L. bulgaricus*, *Lactococcus lactis*, *Streptococcus thermophilus*	18–26 years old, road cyclists (*n* = 26) 4 months, 1 capsule/day	Aerobic capacity GI permeability Inflammatory response	Probiotic supplementation resulted in an increase in aerobic capacity and a decrease in selected markers of GI permeability. Additionally, the circular levels of proinflammatory cytokines and TOS were significantly reduced	[98••]
5.0 × 10^9^ CFU/day multi-strain *B. lactis* W52, *Levilactobacillus brevis* W63, *L. casei* W56, *L. lactis* W19, *L.c. lactis* W58, *L. acidophilus* W37, *B. bifidum* W23, *Ligilactobacillus salivarius* W24	20–60 years old, long-distance runners (*n* = 66) 3 months, 2 capsules/day	GI symptoms	Probiotic supplementation resulted in an improvement in general health and a decrease in the incidence of constipation	[100]
3.0 × 10^11^ CFU/day *Lactiplantibacillus. plantarum* TWK10 Heat-killed *L. plantarum* TWK10	20–30 years old, healthy individuals but not professionally trained (*n* = 53) 6 weeks, 3 capsules/day	Aerobic capacity Fatigue indicators Inflammatory response	Probiotic supplementation improved exercise performance by reducing fatigue indicators and increasing aerobic capacity. NLR decreased in the group that received only heat-killed *L. plantarum* TWK10	[114••]
≥ 5.0 × 10^10^ CFU/day *B. breve* Bif195	18–50 years old, healthy individuals trained in endurance sports (*n* = 57) 6 weeks, 2 capsules/day	GI symptoms	Probiotic supplementation had no effect on exercise-induced intestinal permeability, intestinal integrity, or GI symptoms	[115]
3.0 × 10^10^ CFU/day *L. casei*	19–22 years old, badminton players (*n* = 30) 6 weeks, 80 mL commercial drink/day	Aerobic capacity Psychological state	Probiotic supplementation reduced stress and anxiety, and increased aerobic capacity. However, no significant changes were observed in speed, strength, leg power, and agility	[116]
1.5 × 10^10^ CFU/day multi-strain *L. helveticus* Lafti L10, *B. animalis* ssp. lactis Lafti B94, *Enterococcus faecium* R0026, *B. longum* R0175, *Bacillus subtilis* R0179	19–40 years old, male elite cyclists (*n* = 27) 90 days, 1 capsule/day	GI symptoms Aerobic capacity Inflammatory response	Probiotic supplementation reduced the incidence of nausea, belching, and vomiting at rest and GI symptoms during training. However, no significant difference was observed in aerobic capacity and inflammatory markers	[99]
5.0 × 10^9^ CFU/day multi-strain *L. acidophilus-*LB-G80, *L. paracasei-*LPc-G110, *L. subp. lactis*-LLL-G25, *B. animalis* subp. lactis*-*BL-G101, *B. bifidum-*BB-G90	Healthy male marathon runner (*n* = 14) 30 days, 2 g/day	Upper respiratory tract Inflammatory response	Probiotic supplementation reduced the incidence, number, and severity of URTI symptoms by modulating the immune response	[109]
6.0 × 10^10^ CFU/day *Ultrabiotic 60* and 250 mg SBFloractiv™ *L. rhamnosus*, *L. casei*, *L. acidophilus*, *L. plantarum*, *L. fermentum*, *B. lactis*, *B. bifidum*, *S. thermophilus*, *Saccharomyces boulardi*	Male elite rugby player (*n* = 19) 17 weeks, 1 blend/day	Fatigue indicators Sleep quality	Probiotic supplementation improved sleep quality and reduced perceived muscle soreness	[103]
5.0 × 10^9^ CFU/day multi-strain *B. lactis* W52, *L. brevis* W63, *L. casei* W56, *L. lactis* W19, *L. lactis* W58, *L. acidophilus* W37, *B. bifidum* W23, *L. salivarius* W24	Long-distance runners (*n* = 66) 3 months, 2 capsules/day	Muscle mass Aerobic capacity	Probiotic supplementation increased lean body mass, total muscle mass, aerobic capacity, minute ventilation, breathing reserve, and exercise capacity only in men runners	[117]
2.5 × 10^10^ CFU/day multi-strain *L. acidophilus* CUL60, *L. acidophilus* CUL21, *B. bifidum* CUL20, *B. animalis* subsp. lactis CUL34	Marathon runners (*n* = 24) 28 days, 1 capsule/day	Fatigue indicators	By analyzed by untargeted metabolomics, probiotics had shown a potentially protective effect on marathon-induced metabolic pertubations	[66••]
1.0 × 10^10^ CFU/day multi-strain *B. animalis.* subsp. lactis, *L. acidophilus*	30–45 years old, male marathon runners (*n* = 27) 30 days, 1 sachet/day	Upper respiratory tract Immunity	Probiotic supplementation did not affect the incidence and severity of URTI symptoms. However, it maintained the total number of CD8 + T cells and effector memory population	[110]
5.0 × 10^9^ CFU/day *B. subtilis* DEE11	Division I female athletes (*n* = 23) 10 weeks, 1 capsule/day	Physical performance	Post-exercise probiotic supplementation reduced body fat percentage but had no effect on physical performance	[105]
1.5 × 10^10^ CFU/day *B. longum* subsp. Longum OLP-O1	20–30 years old, long-distance runners (*n* = 21) 5 weeks, 3 capsules/day	Physical performance	Probiotic supplementation did not affect body composition, but significantly increased physical performance during exercise	[118]
1.0 × 10^10^ CFU/day *L. plantarum* 299v	Female iron-deficient athletes (*n* = 53) 12 weeks, 1 capsule/day	İron bioavailability Aerobic capacity Upper respiratory tract	Probiotic supplementation increased plasma iron levels but did not affect aerobic capacity. There was also an association between probiotic supplementation and a reduced incidence of URTI	[104]
2.5 × 10^10^ CFU/day multi-strain *L. acidophilus* (CUL60), *L. acidophilus* (CUL21), *B. bifidum* (CUL20), *B. animalis* subsp. lactis (CUL34)	Male cyclist (*n* = 7) 4 weeks, 1 capsule/day	Carbohydrate bioavailability Carbohydrate metabolism	Probiotic supplementation improved glucose absorption but did not significantly affect muscle and liver glycogen oxidation	[101]
2.0 × 10^9^ CFU/day *B. coagulans* Unique IS-2	18–25 years old resistance-trained men (*n* = 70) 60 days, 20 g powder/day	Amino acid bioavailability Physical performance	Probiotic supplementation improved the absorption of BCAAs and enhanced exercise performance by improving lower body muscle strength	[102]
1.0 × 10^10^ CFU/day multi-strain *L. paracasei* LP-DG® *L. paracasei* LPC-S01	18–35 years old, physically active men (*n* = 15) 2 weeks, 1 sachet/day	Amino acid bioavailability	Probiotic supplementation improved the absorption of total, essential, and branched-chain amino acids	[89]
1.2 × 10^9^ CFU/day multi-strain *B. animalis* subsp. lactis BB-12, *L. bulgaricus* *S. thermophilus*	Young divers (*n* = 21) 8 weeks, 100 g yoghurt/day	Physical performance Psychological state	Probiotic supplementation decreased cognitive state and somatic state anxiety score and enhanced exercise performance. There is no control group in this study	[119]
3.0 × 10^10^ CFU/day *L. plantarum* PS128	Triathletes (*n* = 20) 4 weeks, 2 capsules/day	Physical performance Amino acid bioavailability Inflammatory response	Probiotic supplement alleviated inflammation after intense exercise, improved the absorption of BCAAs, and enhanced exercise performance	[12]
2.5 × 10^10^ CFU/day multi-strain *L. acidophilus* CUL60, *L. acidophilus* CUL21, *B. bifidum* CUL20, *B. animalis* subsp. lactis CUL34	Marathon runners (*n* = 19) 4 weeks, 1 capsule/day	GI symptoms	Probiotic supplementation reduced the incidence and severity of GI symptoms	[120]
2.0 × 10^8^ CFU/day *L. salivarius* UCC118	18–45 years old, trained endurance athletes (n:7) 4 weeks, 1 capsule/day	GI permeability	Probiotic supplementation reduced exercise-induced small intestinal permeability of sucrose	[121]
6.0 × 10^10^ CFU/day multi-strain *L. acidophilus*, *L. lactis*, *L. casei*, *B. longum*, *B. bifidum*, *B. İnfantis*	19–26 years old, healthy sedentary men (*n* = 41) 12 weeks, 2 sachets/day	Immunity	Total lymphocytes and leukocytes, T lymphocytes, helper and cytotoxic T cells, B lymphocytes, and natural killer cell counts were not significantly affected by probiotic supplementation	[122]
6.0 × 10^10^ CFU/day multi-strain *L. acidophilus*, *L. lactis*, *L. casei*, *B. longum*, *B. bifidum*, *B. İnfantis*	19–26 years old, healthy sedentary men (*n* = 41) 12 weeks, 2 sachets/day	Physical performance	Combination of probiotic supplementation and exercise improved physical performance by increasing muscle strength in both legs	[123]
1.0 × 10^9^ CFU/day *B. longum* 35624	Division I female swimmers (*n* = 20) 6 weeks, 1 capsule/day	Physical performance	Probiotic supplementation did not result in a significant difference in concentric/eccentric force generation and overall vertical jump height, as well as in aerobic and anaerobic swimming performance tests	[106]
1.0 × 10^9^ CFU/day *B. subtilis* DE111	Division I male baseball players (*n* = 25) 12 weeks, 1 capsule/day	Physical performance	Probiotic supplementation did not result in any difference in strength, performance, and muscle thickness of the players	[107]

*BCAA* Branched-Chain Amino Acids, *CFU *Colony Forming Units, *GI* Gastrointestinal, *TOS* Total Oxidant Status, *URTI* Upper Respiratory Tract Infections

Intense exercise can induce GI damage, causing GI symptoms, such as abdominal pain, diarrhea, and blood in the stool [98••]. Therefore, intense exercise-induced GI damage has been reported to reduce macronutrient absorption [85, 86]. Multi-strain probiotic supplementation has reduced GI symptoms in intensely trained athletes such as long-distance runners and road cyclists [99, 100]. Additionally, probiotic supplementation has been demonstrated to enhance intestinal macronutrient absorption in a few randomized controlled trials (RCTs) [101, 102]. Jäger et al. (2020) reported that multi-strain probiotic supplementation increased the absorption of plant proteins in the diet of physically active men [89]. Vegetable proteins contain low amounts of BCAAs. Among BCAAs, especially leucine is of high importance for muscle protein turnover. Huang et al. (2019) reported that *L. plantarum* PS128 supplementation improved BCAA absorption after intense exercise and thus maintained exercise performance by increasing muscle power [12]. This finding was also supported by Tarik et al. using *Bacillus coagulans* Unique IS-2 support [102].

Probiotic supplementation can improve physical performance by increasing muscle protein turnover, muscle strength and endurance, fatigue indicators, and aerobic capacity. *Lactobacillus* and *Bifidobacterium* strains can ameliorate exercise-induced fatigue indicators [66••, 103]. Furthermore, multi-strain probiotic supplementation has been reported to increase maximal oxygen uptake and decrease maximal heart rate in road cyclists [98••]. Conversely, Axling et al. (2020) conducted a study which revealed that *L. plantarum* 299v had no impact on aerobic capacity, which was not dissimilar to the effect observed with iron supplementation [104]. The study results may have differed according to the protocols, such as the dose, composition, and test time of probiotics, assessment methods of aerobic capacity, and target population. In addition, some athletes with a lower VO2_max_ can compensate for their performance by using a higher ratio of VO2_max_ to obtain similar oxygen uptake during a race [97].

Currently, the relationship between probiotic supplementation and physical performance in athletes has received increasing attention from researchers. Despite promising findings, a few researchers have shown that probiotics do not affect the physical performance of athletes [105–107]. These current studies were mostly conducted using a single-strain probiotic supplement or *B. subtilis* DE111 supplement. In addition, physical performance measurements have been associated with aerobic capacity in some studies. Aerobic capacity is recognized as an important component of physical performance, and its measurement has almost become routine in the physiological testing of elite athletes [108]. However, physical performance is associated with a combination of several factors, such as type I muscle fibers, glycogen storage capacity, anaerobic power, and aerobic capacity [97]. Consequently, these differences in the evaluation of the results can lead to heterogeneous findings regarding physical performance. It is important to conduct further research to understand the relationship between probiotics and physical performance and provide clearer inferences.

Intense exercise increases intestinal permeability and endotoxemia by causing GI disturbances. This causes an increase in plasma LPS levels in the blood, thus increasing the secretion of proinflammatory cytokines, such as IL1, IL6, and TNF-α, from monocytes [47, 48]. Probiotic supplementation may alleviate the increased inflammatory response after exercise. In a meta-analysis, probiotic supplementation was found to reduce proinflammatory cytokine levels in elite athletes [34•]. Additionally, intense exercises temporarily suppress the immune response and, as a result, increase the risk of infections such as URTIs [46]. Limited current studies with a few participants are available, showing the effect of probiotics on URTI symptoms in athletes. These studies provide conflicting findings regarding the incidence and severity of URTI symptoms [109, 110]. In a meta-analysis, probiotic supplementation had no effect on the number of days of illness or mean number or duration of URTI episodes. However, single-strain probiotics, in particular, have been reported to reduce the total symptom severity score of URTIs [78•]. In this current review, studies investigating the effect of probiotics on URTI were conducted using multi-strain supplementation.

Studies demonstrating the effects of probiotic supplementation on sports performance either, directly or indirectly, present conflicting findings. These discrepancies are thought to be due to different methodologies, such as supplementation type (single or multi-strain) and timing [111, 112]. Factors such as dose of probiotic used, duration of the study, and the evaluation of the data are also likely to affect the results of the studies [98••]. Additionally, information about the microbiome and dietary patterns of athletes has not been investigated in most current studies, which may have caused the findings to be different.

## Conclusion and Future Perspectives

In conclusion, in this review, we consider some potential effects of probiotics such as a decrease in intestinal permeability, decrease in inflammation, decrease in symptoms of upper respiratory tract infections, increase in aerobic capacity, and modulation of immune response. Accordingly, probiotics may be recommended for individuals who exercise. However, the exclusion of information about the microbiome of the athletes and their diets at the beginning of current studies limits the assessment of the results obtained. Additionally, it is difficult to determine direct evidence between exercise-induced impairments in cytokine secretion, intestinal barrier function, and immune response and improvements after probiotic supplementation in these studies. Therefore, well-designed interventional studies are needed to clarify the effects of probiotic supplementation on sports performance. These studies should also evaluate different markers of bowel barrier function, inflammation, and aerobic capacity. Additionally, it may be important to focus on how probiotics alone or in combination with prebiotics can affect the exercise performance of athletes. Further studies are required to draw firm conclusions, as most studies have important methodological limitations such as study design, variation in populations, strain types used, and criteria used to evaluate performance.

## Data Availability

The data that support the findings of this study are available from the corresponding author.
